# Research of Distorted Vehicle Magnetic Signatures Recognitions, for Length Estimation in Real Traffic Conditions

**DOI:** 10.3390/s21237872

**Published:** 2021-11-26

**Authors:** Donatas Miklusis, Vytautas Markevicius, Dangirutis Navikas, Mindaugas Cepenas, Juozas Balamutas, Algimantas Valinevicius, Mindaugas Zilys, Inigo Cuinas, Dardan Klimenta, Darius Andriukaitis

**Affiliations:** 1 Department of Electronics Engineering, Kaunas University of Technology, Studentu St. 50-438, LT-51368 Kaunas, Lithuania; donatas.miklusis@ktu.edu (D.M.); vytautas.markevicius@ktu.lt (V.M.); dangirutis.navikas@ktu.lt (D.N.); mindaugas.cepenas@ktu.lt (M.C.); juozas.balamutas@ktu.lt (J.B.); algimantas.valinevicius@ktu.lt (A.V.); mindaugas.zilys@ktu.lt (M.Z.); 2 Department of Signal Theory and Communications-atlanTTic Research Center, University of Vigo, 36310 Vigo, Spain; inhigo@uvigo.es; 3 Faculty of Technical Sciences, University of Pristina in Kosovska Mitrovica, Kneza Milosa St. 7, RS-38220 Kosovska Mitrovica, Serbia; dardan.klimenta@pr.ac.rs

**Keywords:** road traffic monitoring, vehicle speed estimation, vehicle length estimation, AMR type magnetic field sensor, triple-axis accelerometer, cross-correlation, threshold based method

## Abstract

Reliable cost-effective traffic monitoring stations are a key component of intelligent transportation systems (ITS). While modern surveillance camera systems provide a high amount of data, due to high installation price or invasion of drivers’ personal privacy, they are not the right technology. Therefore, in this paper we introduce a traffic flow parameterization system, using a built-in pavement sensing hub of a pair of AMR (anisotropic magneto resistance) magnetic field and MEMS (micro-electromechanical system) accelerometer sensors. In comparison with inductive loops, AMR magnetic sensors are significantly cheaper, have lower installation price and cause less intrusion to the road. The developed system uses magnetic signature to estimate vehicle speed and length. While speed is obtained from the cross-correlation method, a novel vehicle length estimation algorithm based on characterization of the derivative of magnetic signature is presented. The influence of signature filtering, derivative step and threshold parameter on estimated length is investigated. Further, accelerometer sensors are employed to detect when the wheel of vehicle passes directly over the sensor, which cause distorted magnetic signatures. Results show that even distorted signatures can be used for speed estimation, but it must be treated with a more robust method. The database during the real-word traffic and hazard environmental condition was collected over a 0.5-year period and used for method validation.

## 1. Introduction

With the ongoing rise of vehicle number in the streets, the road traffic sector requires intelligent systems to mitigate congestions by monitoring and controlling the traffic. Adaptive traffic light systems, for streets with volatile traffic direction, are already a must-have thing in modern city life. The first step for this kind of system is to collect useful road traffic information: vehicle volume, speed, length, type and direction [1,2,3]. There are a number of vehicles sensing technologies from simple inductive loops to complex microwave radars and high-speed cameras [4,5,6,7,8,9,10,11,12], and all of them have unique advantages and drawbacks.

This research aims to explore practical traffic classification possibilities, based on data collected during real-life traffic conditions, using a single magnetic AMR sensor hub. The key parameter for vehicle classification is length, whose estimation is dependent on speed measurement. In previous works, it was noticed that using the cross-correlation method to estimate speed produces a high error due to uneven magnetic signals. In this research, the authors identify the main error source and also propose a correction technique. In addition, a novel approach to estimate vehicle length is presented. A great deal of effort has been paid to collect a unique dataset over the past half year and to test proposed methods.

## 2. Relate Works

A variety of sensing techniques are used for increasing road traffic safety. One approach is based on equipping vehicles [13,14,15] with new sensors and another by upgrading road infrastructure with traffic monitoring systems, from inductive loops to microwave radars and optical fibers [4,5,6,7,8,9]. Although many methods can be used for intelligent traffic systems, there are few key technologies widely accepted as reliable: surveillance camera systems, inductive loops and magnetic field sensors. Lately, extensive works have been put on developing intelligent traffic monitoring stations based on magnetic sensors [7,9]. Well-known and mature inductive loop technology were compared with magnetic field sensors in [2]. As a main drawback, the authors identified the installation process and maintenance. Although both technologies require temporary closure of the road, magnetic sensors are much smaller and cause significantly less protrusion to the road pavement. Furthermore, magnetic sensors provide signals with a rich amount of feature points and could be used for classification.

The authors of [16] investigated a vehicle classification approach with signals from a magnetic sensor, placed on roadside 60 cm from passing vehicles. Four types of vehicles were analyzed: sedan, van, truck and bus. The authors proposed to use Mel Frequency Cepstral Coefficients (MFCC) as a feature to characterize magnetic signatures. Furthermore, Dynamic Time Wrapping (DTW) algorithm was employed to select efficient training samples and filter out distorted ones. The proposed classifier algorithm categorized vehicles in five groups, but neither length nor speed estimation results were presented.

Similar approaches are discussed in the papers [17,18]. Firstly, a dataset of magnetic signatures was collected and organized into four main groups: sedan, van, bus and trucks. Secondly, after digital signal processing and feature extraction, the classifier was trained based on these signals. As a result, the presented methods did not provide absolute value of vehicle length/height estimation, but rather just assigned vehicles to a particular group. As compared in the paper [17], their overall accuracy of classification into seven types was 90.3%. For speed estimation, the authors proposed a different method. Since only one magnetic field sensor is employed in the paper [18], speed was estimated by assigning unknown signature to one of the four vehicle types and applying coefficients of particular model. The authors claim that only 8% of the estimated speed errors exceeds 10 km/h, and 80% of errors are lower than 4 km/h. In comparison, Kafeng Wang [17] chose to place two laterally displaced sensors along the road and estimated the speed of vehicle through maximizing the correlation between the signals. Consecutively, this information was used for length estimation and assignment into the group. As it is explained in both papers, filtering plays an important role for signal processing. In the first paper, a 5 Hz low pass filter was applied, whereas in the second, Butterworth low (>40 Hz) and high pass filters (<10 Hz) were employed as a bandpass.

In the recent years, wireless sensors have received significant attention in automated systems [19,20,21]. The authors of [19] proposed a sensing node consisting of two magnetic field sensors on the roadside and one on the road. Magnetic readings of the z-axis from the multiple sensors were used for vehicle type estimation and classification. In [20], vehicle classification is accomplished only from one node sensor. The authors shared future plans to collect a large dataset and arrange data into four main groups. Once a new signature is detected, a classification algorithm based on Euclidean distance calculation will assign each vehicle to the closest distance sample category.

Networks of sensing systems are necessary for designing traffic jam avoidance, road safety and traffic surveillance systems. A very attractive self-powered unit idea is explored in papers [22,23].

## 3. Problems

During the previous research [9], it was noted that at certain conditions speed estimation method from the pair of magnetic signatures gave highly faulty results. After the analysis, it was deducted that most of the time, incorrect results appeared once the wheel of the vehicle passed very close or directly above the sensor. This kind of distorted magnetic signatures must be identified and treated in a different manner; therefore, a system of magnetic field sensor with accelerometer is presented in the following section.

### 3.1. Distorted Signatures Identification by Accelerometer Signal

**Figure 1:**
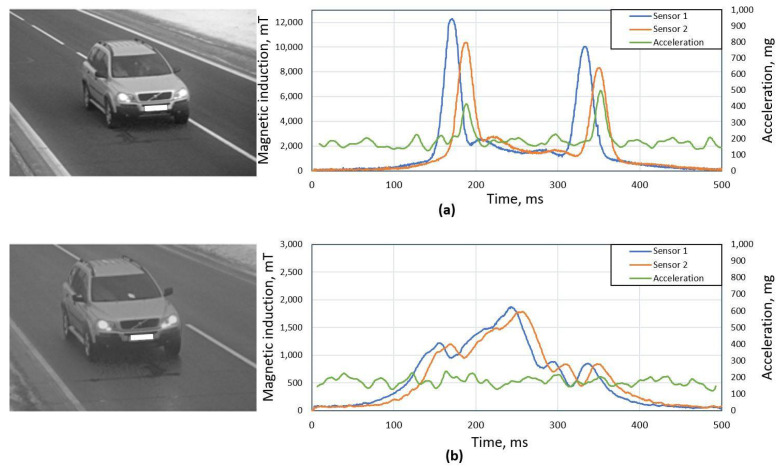
An example of same vehicle passing traffic monitoring station in two ways: (**a**) right side wheels are directly passing over the sensor, (**b**) vehicle driving at the center of sensor hub. In the plots next to the images, temporal data of magnetic signature of two magnetic sensors and accelerometer signal are shown.

In Figure 1, we can see an example of the same vehicle passing a magnetic field sensor by the correct and distorted manner. As it is visible in the Figure 1a plot, distorted magnetic signatures of both sensors have significant peaks at the leading and trailing edges, while in the Figure 1b plot we can see how signals should look like for a particular vehicle if it drives at the center of sensor. Furthermore, in the Figure 1a plot, two peaks of accelerometer signal are clearly visible, which are caused by front and rear tires.

In our application, an STMicroelectronics LIS3DH triple-axis accelerometer is used, and sampling frequency 1 kHz. Since the sensor node is installed at 6 cm depth into the pavement, it is important to guarantee good mechanical interface. The inner sensor housing was filled with epoxy, and during the installation on the road, the sensor was poured over with specialized tar. As a result, accelerometer detects vehicles only if their wheels drive directly above the sensor.

**Figure 2:**
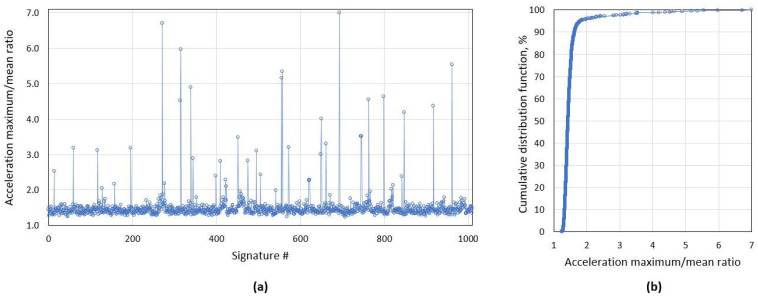
(**a**) Accelerometer maximum/mean value ratio distribution for the current test group of 1000 unique signatures with (**b**) cumulative distribution function (CDF). As it is visible, 95.7% of all cases has a ratio below 2.

Due to outside temperature changes, the mechanical properties of road surface changes and likewise the noise level for accelerometer signal. Therefore, it is desirable to use an adaptive algorithm for vehicle driving over the sensor detection. For signal processing, first, low pass filtering is applied, and as a criterion, it is proposed to use the ratio of accelerometer signal maximum and mean values (Figure 1). In Figure 2, one can see the ratio values for the test group of 1000 randomly selected vehicles, and on the right side the cumulative distribution function for the same criterion. For this test group, 95% of cases ratio is below value acc_ratio = 2, which we use as the threshold value to identify the vehicle which drove directly over the sensor.

**Figure 3:**
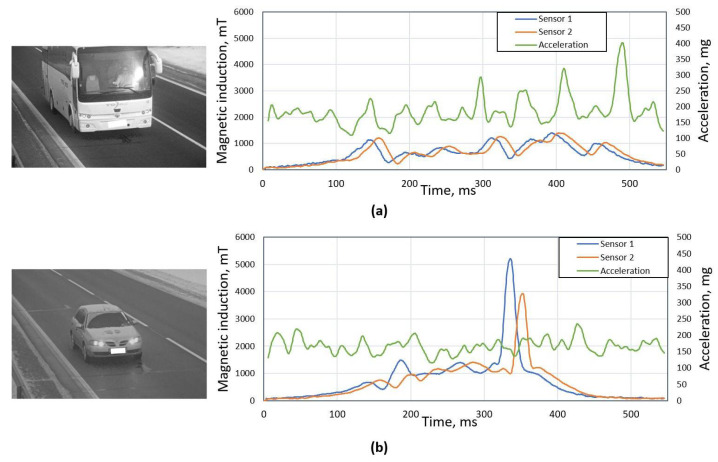
An example of two cases, when accelerometer criterion gives faulty result: (**a**) plot: acc ratio of 2.2 indicates distorted signature, but bus was driving perfectly at the center of sensor and magnetic signatures are not distorted; (**b**) plot: vehicle is clearly driving over the sensor therefore magnetic signatures are distorted, but from the accerometer signal it is not visible (acc_ratio = 1.43).

Although the accelerometer helps to detect distorted signatures, it is not always a sufficient approach, as shown in Figure 3. In the Figure 3a plot, one can see a case that a heavy vehicle produces high level of vibration (acc_ratio = 2.2), which indicates a distorted magnetic signature. Although from the image and magnetic signals, it is clear that it is not a distorted case. An opposite case is shown in the Figure 3b plot, where the accelerometer signal does not indicate anything, although magnetic signatures are highly distorted.

### 3.2. Distorted Signature Detection Based on Feature of Magnetic Signals

As it was explained in the section above, the accelerometer is not sufficient for detecting distorted signatures and an additional method should be employed. It was proposed to explore signals from magnetic sensors.

**Figure 4:**
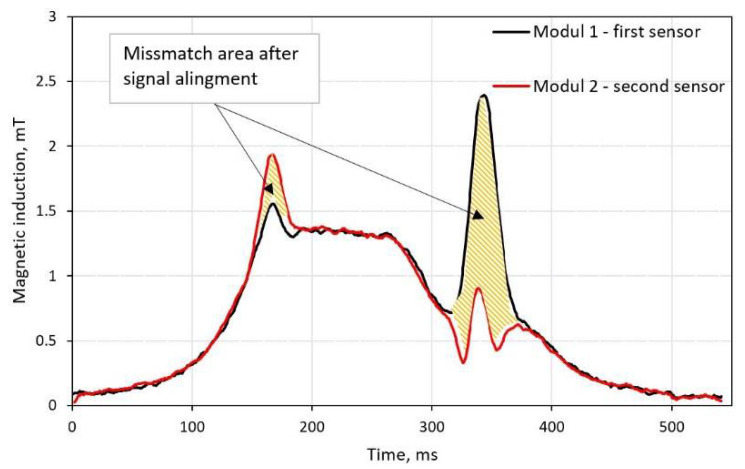
Signal similarity estimated by calculating relative mismatch area between two curvatures. Alignment is based on lag value from the cross-correlation method.

Normally, if the vehicle passes at the center of magnetic sensors, a pair of magnetic signals are identical and just shifted in time (shape and area of curvature should match). In order to detect distorted signatures, one could compare the mismatch area of curvature (Figure 4). Needless to say, signals must be aligned in time, and cross-correlation is the preferable method. It is done by finding a lag of two signals and then subtracting it from the leading signal. In the example below, one can see two aligned magnetic signatures with mismatch area above 10% of the total figure area of the first signal.

**Figure 5:**
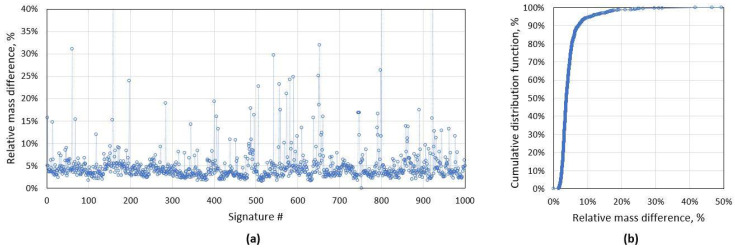
(**a**) The mismatch criteria distribution for the whole dataset of 1000 signatures and (**b**) cumulative distribution function (CDF) for the same data. The average mismatch between two aligned curves is 5%, and 95% of all cases have criterion below 10%.

By applying only the accelerometer criterion, up to 41 distorted signature pairs were detected; by using second mismatch criterion, up to 58 signatures (Figure 5). Twenty-one cases were detected by both methods simultaneously, whereas the rest of cases were detected only by one of the methods. By using two methods, a total of 78 cases were detected, representing 7.8% of distorted signatures in the dataset.

## 4. Method and Materials

**Figure 6:**
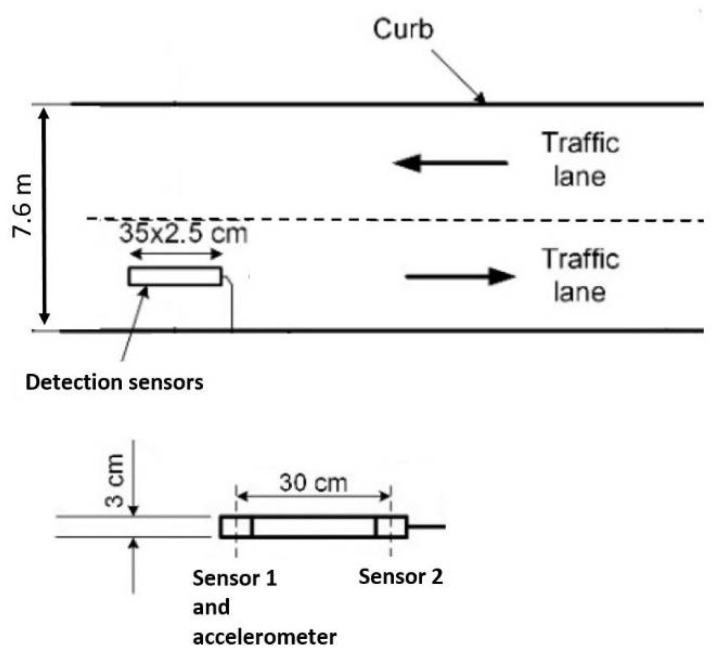
The schematic drawing of the traffic monitoring station. The sensor hub consist of two magnetic field sensors spaced by 30 cm and one accelerometer.

In this paper, we use data collected from a traffic monitoring station, as explained in paper [9]. Additionally, this station was upgraded with accelerometer sensors, which were installed on the same PCB (printed circuit board) with magnetometers (Figure 6). In this system, two STMicroelectronics LIS3MDL AMR type magnetic field sensors are used with sampling frequency of 2 kHz. Two sensors were placed at one space point and their scanning was performed alternately every 0.5 ms; thus, the magnetic field data at a given space point are read at a frequency of 2 kHz. Originally, the sensors record incident magnetic field B in three geometrical axes providing the three corresponding components (Bx, By, Bz). However, for further calculation, we will be using only magnetic signature term, which refers to the magnitude of magnetic field (1).

(1) $$B=\sqrt{Bx+By+Bz},$$

where: B—magnitude of magnetic field, Bx By Bz—three orthogonal magnetic field components.

### 4.1. Speed Estimation Technique for Distorted Signatures

An accurate speed estimation from magnetic signals is the first step to calculate vehicle length and type. In the previous works [3,9,24], it was shown that speed estimation technique based on cross-correlation produces the most accurate results. Therefore, speed is estimated from the pair of magnetic signatures magnitude in this research. Prior to cross-correlation calculation signals are filtered with low pass filter of 100 Hz.

**Figure 7:**
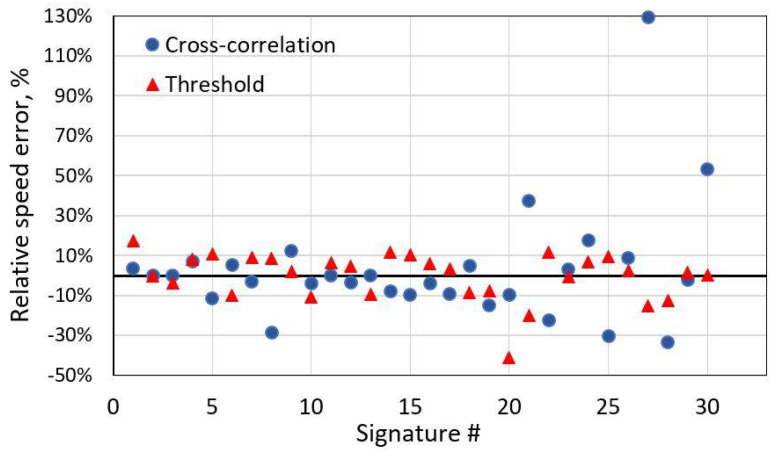
Relative speed error of the test group with distorted signatures with two methods. Relative speed error standard deviation for the cross-correlation method is 30%; for the threshold-based method, it is 12%.

This method works well with similar shape signals. However, it is not the case with distorted magnetic signatures. As depicted below, some distortions could lead to high-speed estimation errors. In these cases, it is more reliable to use a simple threshold-based method to estimate speed. Using this approach, vehicle arrival and departure points in time are detected depending on the amplitude of magnetic signature. As shown in Figure 7, the cross-correlation method with distorted signatures might produce very high errors. On the contrary, a simple threshold-based method is more robust for it. For the distorted test group, standard deviation of relative speed error is 30% for the cross-correlation and only 12% by using the threshold-based method.

### 4.2. Vehicle Lenght Estitmation

One of the most important parameters used for vehicle classification is length. By using magnetic sensors data, it could be estimated by different methods. Hereafter, a comparison of two methods is analyzed:

- Threshold-based method from signature magnitude [24];
- Peak detection method from derivative of magnitude.

The first method is based on the hypothesis that, at the magnetic sensor position, in the presence of vehicle, magnetic magnitude level rises above a certain threshold. In order to minimize the resulting length error, low pass filtering was applied to data and multiple threshold levels were tested. This method works well in laboratory conditions, but not in real life conditions. As it was noted from real traffic data, magnetic signatures amplitude is not a sufficient criterion for vehicle length estimation.

Therefore, a new method based on signal amplitude and shape was proposed. It employs very first/last significant peak detection of 1st derivative of magnetic signature. After studying a huge variety of vehicle magnetic signatures, a few parameters for this method were adopted to detect feature points.

**Figure 8:**
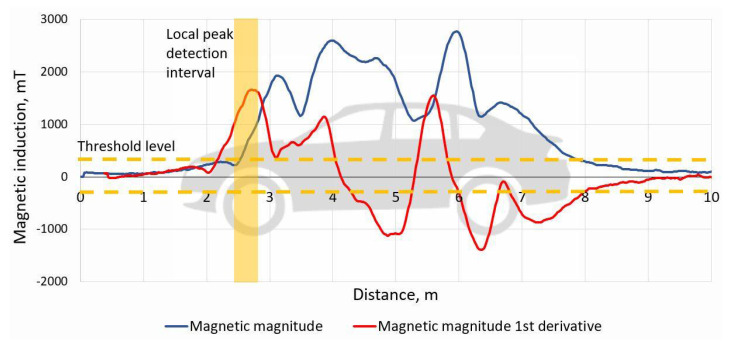
An example of vehicle magnetic magnitude and its 1st derivative signals. According to the above explained algorithm, the time-based signature is converted to a distance-based signal. Peak detection algorithm is employed to locate the very first and the last significant peaks above the threshold level.

Peak detection method is based on the following steps (Figure 8):

1. Convert the time-based signature to distance-based.
  Time array is converted by 2 cm discrete samples, using speed value from cross-correlation method.
2. Calculate the 1st order derivative of the distance-based signature. The derivative calculation step is 0.8 m.
3. Locate the first and the last significant peaks, which represents front and rear of the vehicle.
4. Estimate the gap between the first/last peaks vehicle length.

Peaks detection algorithm works by locating the very first and the last significant peaks, which need to fulfill two conditions: peak must be above amplitude threshold level and must be a local peak in 0.3 m range.

## 5. Result and Accuracy

The methods were tested with two different datasets. The first dataset consisted of 300 unique pairs of signatures of different type of vehicle, from standard length passenger cars to long trucks (>18 m). The model and make of each vehicle were identified from pictures and reference length was extracted from public database.

The second dataset consisted of 14 unique vehicles with multiple passes. Each vehicle was detected more than 14 times, which sums up to a total of 243 pairs of signatures. It was done by applying license plate recognition to the images and filtering according to it.

It is worth noting that the data was collected from monitoring station under real traffic conditions. Data collection was performed in approximately a half-year period. Since the sensor hub is located in the middle of a one-way lane of a two-lane road without any physical traffic confinement, vehicles could pass over the sensor not necessarily at the center.

### 5.1. Vehicle Length Estimation Results with the First Dataset

Different parameters were compared to analyze the performance of the methods: mean absolute error (2), number of outlier and range of interquartile. It is desirable to minimize MAE (Mean Absolute Error), but at the same time it is important to avoid a high number of outliers.

(2) $$\mathrm{MAE}=\frac{1}{M}\overset{M}{\underset{m=1}{\sum }}\left|L-L_{r}\right|,$$

where: M—number of vehicles in dataset (300 samples), L—estimated length, $L_{r}$—reference length from public database, m—vehicle number in the dataset.

**Figure 9:**
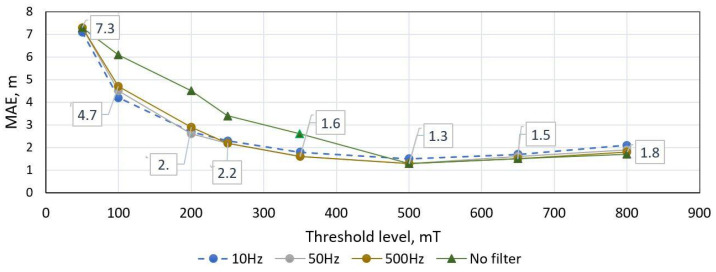
Threshold-based length estimation method MAE score versus low pass filtering (fc) and threshold value. Dataset of 300 unique vehicle used.

**Figure 10:**
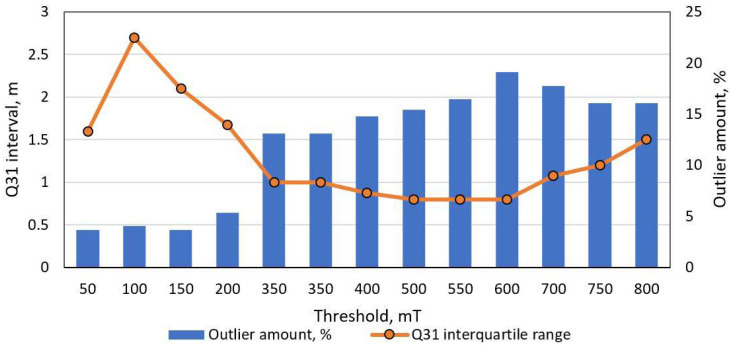
Threshold-based length estimation method performance versus threshold level. The orange line depicts interquartile range while columns indicate number of outliers. Dataset of 300 unique vehicle used, filtered with 50 Hz LPF.

In order to tune methods for general purpose, values of parameters were estimated in the trial method. First, the threshold-based method was tested with different low pass filter cut-off frequencies (fc) and threshold values (Figure 9 and Figure 10). As it is visible in charts below, an initial filtering is necessary to remove noise, but fc lower than 500 Hz does not help to reduce error. Furthermore, it is visible that minimum MAE (1.3 m) is reached at threshold level 500 mT.

**Figure 11:**
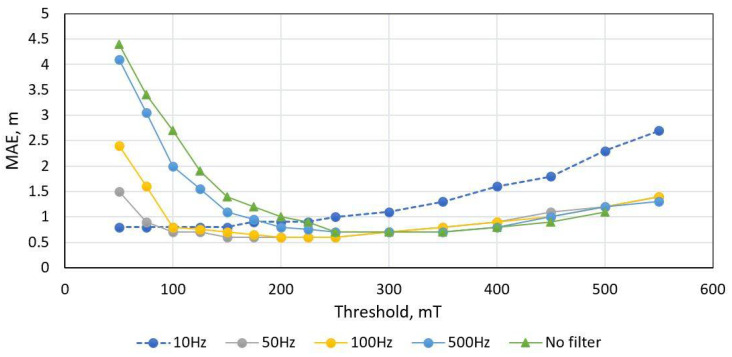
Peak-detection length estimation method MAE score versus low pass filtering (fc) and threshold value. Dataset of 300 unique vehicle used.

**Figure 12:**
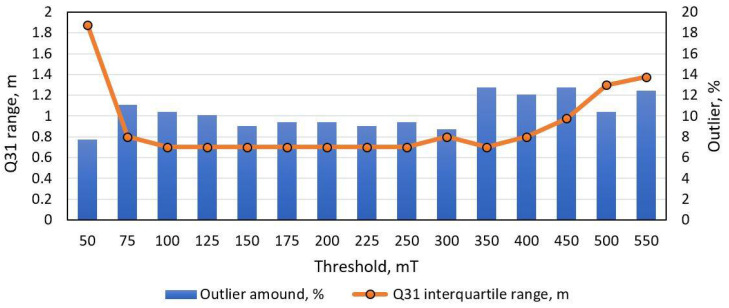
Peak-detection length estimation method performance with dataset of 300 unique vehicle. The orange line depicts error interquartile range, while blue columns indicate the number of outliers.

The proposed peak detection method was also tested with the dataset and the results are shown in Figure 11 and Figure 12. As presented, low pass filtering has a significant influence on the error, but filtering below 50 Hz also filters out signature features, which causes increased error. Since this method is based on peak detection over a threshold, it produces the same result with threshold level in certain range. The minimum MAE is obtained with 50 Hz filtering in threshold range of 100–2500 mT. It is visible that, at threshold level of around 150 mT, the interquartile range and the number of outliers stay the same.

### 5.2. Vehicle Length Estimation Errors with the Second Dataset

**Figure 13:**
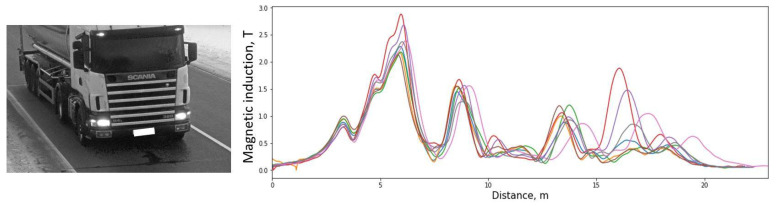
An example of a group of magnetic signatures produced by the same vehicle. Signatures rescaled according to the speed and aligned by the first peak.

The second dataset contains multiple signatures of the same vehicle. In an ideal case, the same vehicle should produce identical signatures and shall result in the same estimated length. However, in real traffic conditions, the magnetic signature varies in shape. In Figure 13, the multiple signatures of the same truck with a tank are presented. Signals are rescaled according to speed and aligned by the first significant peak.

In Table 1, length estimation results of the proposed method are summarized. In this table, vehicles of different length are presented, from short passenger cars to buses and a truck. With all these different types of vehicles, RMSE does not exceed 1 m and mean standard error is 0.42 m. It is also visible that for the vehicle shown in Figure 13, mean estimated length is 15.6 m with mean error of 0.47 m.

## 6. Discussion

Vehicle classification systems are continuously improving. A number of papers are published every year, aiming to propose new, better and cost-effective methods. Although authors claim significant classification results, proposed methods are tuned for specific test groups and simple performance metrics are missing, which makes it difficult to compare with other algorithms.

In order to classify vehicles, length is the first parameter to consider. Therefore, we aim to analyze possibilities and limitations of vehicle length estimation based on magnetic field sensors. Since this study is based on data collected within real life traffic conditions, the distorted signal problem was tackled first. Furthermore, as was shown in the Related Works section, current state-of-the-art methods jump over intermediate results (e.g., vehicle length) and analyze only final classifier results. Although, for most of applications vehicle type is more important than its length, here we wanted to analyze accuracy and limitation of length estimation method from magnetic signatures. Furthermore, speed estimation is a necessary part for obtaining the length. Needless to say, speed has to be obtained from same magnetic signals. Therefore, estimated vehicle length error depends on two factors: error of speed and error of length estimation method itself.

A novel vehicle length estimation method was tested with two different datasets. As shown in the Results section, the simple threshold based method minimum MAE score is 1.3 m, while the number of outliers is above 15% and it highly depends on the threshold level. Our proposed method gives a minimum MAE score of 0.6 m and the number of outliers is below 10% using the same dataset. Furthermore, as visible in Figure 12, the value of interquartile range Q31 and number of outliers are much less dependent on the threshold level. It is an important feature of the proposed method, since it shows that this method is more robust and could be easily adapted to any new dataset. Furthermore, in Table 1 one can see the length estimation error for multiple signatures of the same vehicle. We can see that STD (Standard Deviation Error) for the same vehicle varies from 0.1 m to 0.6 m, while the RMSE (Root Mean Squared Error) is 0.6 m.

As shown above, distorted magnetic signatures are not an uncommon problem and it requires a clear processing algorithm. We identified that most distortions appear when one of the vehicle wheels passes directly above the AMR sensor. This problem is not well described in the literature, probably due to fact that other authors have filtered out particular distortions or problem did not appear due to a physical barrier in the test set-up [11,17]. We proposed two criteria to identify distorted signatures: the ratio of accelerometer signal maximum and mean values, and the relative mismatch area of a pair of magnetic signatures. It is shown in Figure 2 and Figure 5 that the proposed methods detect different type of distortions and shall be used together. By applying particular criteria, 7.8% of distorted signatures were detected in the randomly collected test dataset.

Reliable traffic flow parametrization using AMR type sensors is not a trivial task. As was shown, real traffic conditions require additional sensing channels to distinguish distorted signatures and apply different estimation methods. Furthermore, the magnetic signature of a vehicle highly depends on driving trajectory—the same vehicle might produce very different signals. Therefore, an array of sensor nodes might be used in future works.

## Figures and Tables

**Table 1:** Length estimation method result with the second dataset.

Nr, #	Vehicle	Number of Signatures, #	Real Length, m	Mean Estimated Length, m	RMSE, m	STD, m
1	Truck with a tank	16	15.3	15.6	0.7	0.5
2	MB Sprinter1	17	7.3	7.7	0.7	0.6
3	Isuzu bus	14	9.1	9.2	0.6	0.6
4	Audi A6	16	4.1	5.0	1.0	0.3
5	Audi A4_1	16	4.5	4.3	0.3	0.3
6	VW Passat	18	4.6	4.8	0.6	0.6
7	Nissan Primera	17	4.7	4.2	0.5	0.4
8	MB Sprinter2	20	7.3	8.0	0.9	0.3
9	Audi A4_2	20	4.5	4.2	0.2	0.1
10	MB GLE	15	4.8	4.5	0.5	0.5
11	VW Sharan	17	4.9	4.5	0.6	0.6
12	VW Touran	16	4.4	4.3	0.4	0.4
13	VW Transporter	14	5.3	5.2	0.3	0.3
14	BMW serie 5	27	4.8	3.8	1.0	0.5
	Total:	243			0.6	0.42

## Data Availability

Not applicable.
